# Supplementary material for: Morphological State Transition Dynamics in EGF-Induced Epithelial to Mesenchymal Transition
Source: J Clin Med. 2019 Jun 26;8(7):911. doi: 10.3390/jcm8070911 (PMC6678216; doi:10.3390/jcm8070911)
Supplement: Supplementary file 1 [file jcm-08-00911-s001.pdf]

Supplementary Information

Morphological State Transition Dynamics in EGF-induced Epithelial to Mesenchymal Transition

Vimalathithan Devaraj, Biplab Bose

Department of Biosciences and Bioengineering, Indian Institute of Technology Guwahati, Guwahati, India 781039

S1. Cell State Transition Model:

In our experiments, we have observed three distinct cell-types based on morphology: cobble (CB), elongated spindle (ES) and circular (CR). Each of these cell types is considered as an individual cell state. In our cell state transition model, we have assumed that a cell can transit from one state to another. The total number of cells in our experimental system varies with time as cells divide and die. Therefore, we have considered death and birth of cells. To accommodate cell death in our model, an absorbing state called dead state (DD) is considered. The state transition model is graphically represented in the Supplementary Figure S1.

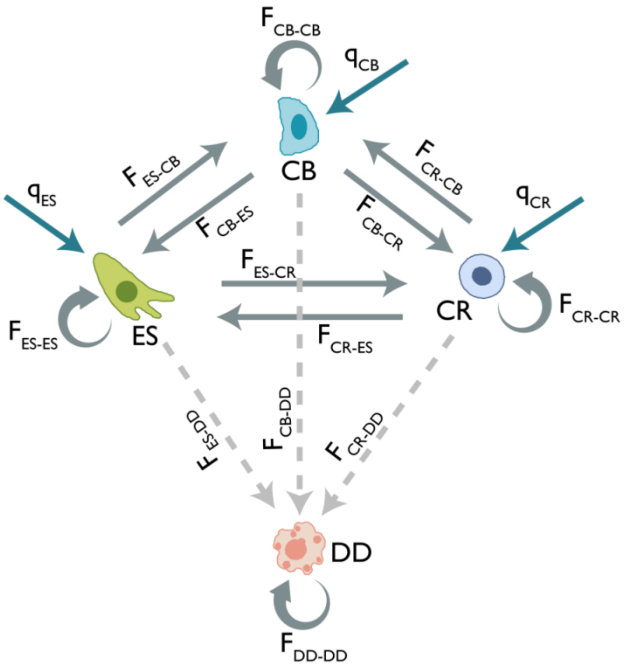

Supplementary Figure S1: State Transition Model. A live cell can be in any of the three morphological states CB, ES, and CR. Any cell can die and move to the dead state (DD). Birth of new cell in a particular state is shown by the arrow without source. $F_{i\text{-}j}$ represents the fraction of cells in i^{th} state moving to the jth state in a time interval. q_i is the fraction of cells in i^{th} state dividing in a time interval.

Mathematically this state transition model is represented by the following set of conservation equations for a time interval [t, $t+\Delta t$]:

$$N_{CB}(t+\Delta t) = N_{CB}(t)\times F_{CB-CB}(t) + N_{ES}(t)\times F_{ES-CB}(t) + N_{CR}(t)\times F_{CR-CB}(t) + N_{CB}(t)\times q_{CB}(t) \qquad -Eq\ (1)$$

$$N_{ES}(t+\Delta t) = N_{CB}(t)\times F_{CB-ES}(t) + N_{ES}(t)\times F_{ES-ES}(t) + N_{CR}(t)\times F_{CR-ES}(t) + N_{ES}(t)\times q_{ES}(t) \qquad -Eq\ (2)$$

$$N_{CR}(t+\Delta t) = N_{CB}(t)\times F_{CB-CR}(t) + N_{ES}(t)\times F_{ES-CR}(t) + N_{CR}(t)\times F_{CR-CR}(t) + N_{CR}(t)\times q_{CR}(t) \qquad -Eq(3)$$

$$N_{DD}(t+\Delta t) = N_{CB}(t)\times F_{CB-DD}(t) + N_{ES}(t)\times F_{ES-DD}(t) + N_{CR}(t)\times F_{CR-DD}(t) + N_{DD}(t)\times F_{DD-DD}(t) \qquad -Eq(4)$$

$N_i(t)$ and $N_i(t+\Delta t)$ are the total number of cells (live as well as dead) at state i at $time = t$ and $time = t+\Delta t$ respectively; i = *CB, ES, CR, DD*.

$F_{i-j}(t)$ is the fraction of cells of ith state moving to the jth state in the interval [t, $t+\Delta t$]; here, i, j=*CB, ES, CR, DD*.

$q_i(t)$ is the fraction of cells in i^{th} state dividing in the interval [t, $t+\Delta t$]; i = *CB, ES, and CR.*

The system has the following constraints:

a) $0 \le F_{i-j}(t) \le 1$, for i = *CB, ES* and *CR; j = CB, ES, CR* and *DD*.

b) $\sum_{j=CB,ES,CR,DD} F_{i-j}(t) = 1$, for i = *CB, ES, CR*.

c) $F_{DD-j}(t) = 0\ and\ F_{DD-DD}(t) = 1$ for j = *CB, ES, CR*.

d) $0 \le q_i(t) \le 1$, for i = *CB, ES,* and *CR*.

Equation 1-4 can be written in terms of fold change in cell number in a time interval and fraction of cells in each cell state:

$$fd(t) \times f_{CB}(t + \Delta t)$$
$$= f_{CB}(t) \times F_{CB-CB}(t) + f_{ES}(t) \times F_{ES-CB}(t) + f_{CR}(t) \times F_{CR-CB}(t) + f_{CB}(t) \times q_{CB}(t)$$

- Eq (5)

$$fd(t) \times f_{ES}(t + \Delta t)$$
$$= f_{CB}(t) \times F_{CB-ES}(t) + f_{ES}(t) \times F_{ES-ES}(t) + f_{CR}(t) \times F_{CR-ES}(t) + f_{ES}(t) \times q_{ES}(t)$$

- Eq (6)

$$fd(t) \times f_{CR}(t + \Delta t)$$
$$= f_{CB}(t) \times F_{CB-CR}(t) + f_{ES}(t) \times F_{ES-CR}(t) + f_{CR}(t) \times F_{CR-CR}(t) + f_{CR}(t) \times q_{CR}(\mathrm{t})$$

- Eq (7)

$$fd(t) \times f_{DD}(t + \Delta t)$$
$$= f_{CB}(t) \times F_{CB-DD}(t) + f_{ES}(t) \times F_{ES-DD}(t) + f_{CR}(t) \times F_{CR-DD}(t)$$
$$+ f_{DD}(t) \times F_{DD-DD}(t)$$

- Eq (8)

Here,

Fold change in total number of cells (live and dead) in the interval [*t*, *t*+ Δt],

$$fd(t) = \frac{N(t + \Delta t)}{N(t)}$$

$f_i(t)$ and $f_i(t+\Delta t)$ are fraction of cells in the ith state at time *t* and $(t+ \Delta t)$ respectively; *i = CB, ES, CR, DD.*

Summation of Equations 5-8 gives the overall conservation equation of the system,

$$fd(t) = 1 + [f_{CB}(t) \times q_{CB}(t) + f_{ES}(t) \times q_{ES}(t) + f_{CR}(t) \times q_{CR}(t)]$$

- Eq(9)

S2: Estimation of model parameters for the state transition model:

We measured the fraction of cells in different cell states (CB, ES, CR) at different time points (t = 0, 12, 24, 36, 48 and 60 h) by image analysis. We also measured the fraction of dead cells and the fold change in the total cell number (live as well as dead) at each time point. We use the data from these experiments to estimate the model parameters.

S2.1 Estimation of the fraction of cells dividing:

Our experimental observations are at six time points (t = 0, 12, 24, 36, 48, 60 h). From equation 9, we estimate the fraction of cell dividing at each time interval. Equation 9 can be written in matrix form,

$$[fd - 1]_t = [f_{CB} \quad f_{ES} \quad f_{CR}]_t \times \begin{bmatrix} q_{CB} \\ q_{ES} \\ q_{CR} \end{bmatrix}_t$$

In vector notation, this equation is written as

$$K(t) = \boldsymbol{F}(t) \times \boldsymbol{q}(t)$$

We estimated the unknown $\boldsymbol{q}(t)$, through linear least square optimisation. We used Trust-Region-Reflective Algorithm implemented in MATLAB *lsqlin* function [1].

The estimated q_i values are shown in the Supplementary Table S1.

S2. 2 Estimation of fractional flow of cells from one state to other states:

Cell state transition in our experiments is reversible and key signaling processes like phosphorylation of EGFR changes with time. Also, we have not observed any steady state for cell state transition data in the interval of 0 to 60 hr. Therefore, we cannot consider constant flow

rates of cells from one state to another. We estimated the fractional flow of cells from one state to another state for each of the 12 hr intervals.

Eq (5-8) can be written in matrix format as follows,

$$\begin{bmatrix} fd \times f_{CB} \\ fd \times f_{ES} \\ fd \times f_{CR} \\ fd \times f_{DD} \end{bmatrix}_{t+\Delta t} = \begin{bmatrix} F_{CB-CB} + q_{CB} & F_{ES-CB} & F_{CR-CB} & 0 \\ F_{CB-ES} & F_{ES-ES} + q_{ES} & F_{CR-ES} & 0 \\ F_{CB-CR} & F_{ES-CR} & F_{CR-CR} + q_{CR} & 0 \\ F_{CB-DD} & F_{ES-DD} & F_{CR-DD} & 1 \end{bmatrix}_t \times \begin{bmatrix} f_{CB} \\ f_{ES} \\ f_{CR} \\ f_{DD} \end{bmatrix}_t$$

Following the constraint of the model, $\sum_{j=CB,ES,CR,DD} F_{i-j}(t) = 1$, where *i* = *CB* or *ES* or *CR*, we do not need to separately estimate the fractional flow of cells to dead state. Therefore, the above matrix can be reduced to,

$$\begin{bmatrix} fd \times f_{CB} \\ fd \times f_{ES} \\ fd \times f_{CR} \end{bmatrix}_{t+\Delta t} = \left[\begin{bmatrix} F_{CB-CB} & F_{ES-CB} & F_{CR-CB} \\ F_{CB-ES} & F_{ES-ES} & F_{CR-ES} \\ F_{CB-CR} & F_{ES-CR} & F_{CR-CR} \end{bmatrix}_t + \begin{bmatrix} q_{CB} & 0 & 0 \\ 0 & q_{ES} & 0 \\ 0 & 0 & q_{CR} \end{bmatrix} \right] \times \begin{bmatrix} f_{CB} \\ f_{ES} \\ f_{CR} \end{bmatrix}_t$$

This can be written in vector notion as

$$\boldsymbol{C}(t + \Delta t) = [\boldsymbol{A}(t) + \boldsymbol{Q}(\boldsymbol{t})] \times \boldsymbol{B}(t) \qquad \text{- Eq (10)}$$

We have estimated the elements of $\boldsymbol{Q}(t)$ in section S2.1. Now we estimate $\boldsymbol{A}(t)$ of Equation 10, for each 12 hr interval, from the experimental data using parameter optimization. Objective function for this optimization can be written as $\min_{\boldsymbol{A}(t)}[\boldsymbol{e}(t)^{\boldsymbol{T}}\boldsymbol{e}(t)]$ where $\boldsymbol{e}(t)$ is the residual between observed and estimated $\boldsymbol{C}(t+\Delta t)$.

However, for an underdetermined system, such an objective function can cause overfitting and can generate extremely different $\boldsymbol{A}(t)$for each time interval. That would be unrealistic as the cell state transition parameters in two successive time points in a real biological system will not differ drastically.

Following Chiba *et al.* [2] we assume that the difference in each element of $\boldsymbol{A}(t)$ of two consecutive time intervals is small in terms of *L1*-norm of the difference. This allows us to constrain the parameter space and estimate the $\boldsymbol{A}(t)$ for all the time intervals simultaneously.

The objective functions used for estimation of cell state transition parameters are,

Objective function 1 = $\min_{\boldsymbol{A}(t)}(\sum_{t\in T}\sum(\boldsymbol{e}(t)^T\boldsymbol{e}(t)))$

Objective function 2 = $\min_{\boldsymbol{A}(t)}(\sum_{t\in T}\sum(|\boldsymbol{A}(t+\Delta t)-\boldsymbol{A}(t)|))$

Here, T = (0 h, 12 h, 24 h, 36 h, 48 h) and Δt = 12 h.

We used a well-established multi-objective genetic algorithm, NSGA-II, proposed by Deb [3] that is implemented in MATLAB function *gamultiobj*. This was used to minimize both the objective functions simultaneously. We performed 1000 independent optimization, and the best parameter set in each run was decided through Pareto front analysis [4]. The best parameter set from these 1000 optimization runs is considered as the optimized parameter values and used in further analysis. A similar method was used to estimate the transition parameter for the short-time experiment (T = 24, 27, 30, 33, 36 h).

All the estimated parameters are shown in the Supplementary Table S2. As a representative data of the estimation method, the Supplementary Figure S13a shows the Pareto plot for one of the thousand independent runs for estimating the parameters for cells treated with 10 ng/mL of EGF. Supplementary Figure S13b shows the distribution of objective functions for these thousand runs. Supplementary Figure S14 shows the estimated fraction of cells in each cell state from the model using the selected parameter set.

We have visualized the cell state transitions diagrammatically in terms of normalized flux through different state transition paths (only live cell states). Normalized flux for a particular cell state transition path *i-j* in a particular time interval *[t, t+Δt]* is defined as,

$$J_{i-j}(t) = \hat{F}_{i-j}(t) \times \hat{f}_i(t)$$

$\hat{F}_{i-j}(t)$ is the fraction of live cells moving from i^{th} state to j^{th} state at time *t*. *i*, *j* = CB, ES and CR. $\hat{f}_i(t)$ is the fraction of live cells at time *t*. *i* = CB, ES, and CR.

S3. Null model:

In the null model, we assume that the observed changes in the distribution of cells in three morphological states originate solely from cell division and cell death and there is no transition from one live cell state to another live cell state.

The model for this is just a reduced version of our full model,

$$fd(t) \times f_{CB}(t + \Delta t) = f_{CB}(t) \times F_{CB-CB}(t) + f_{CB}(t) \times q_{CB} \quad \text{- Eq (11)}$$

$$fd(t) \times f_{ES}(t + \Delta t) = f_{ES}(t) \times F_{ES-ES}(t) + f_{ES}(t) \times q_{ES} \quad \text{- Eq (12)}$$

$$fd(t) \times f_{CR}(t + \Delta t) = f_{CR}(t) \times F_{CR-CR}(t) + f_{CR}(t) \times q_{CR} \quad \text{- Eq (13)}$$

$$fd(t) \times f_{DD}(t + \Delta t)$$
$$= f_{CB}(t) \times F_{CB-DD}(t) + f_{ES}(t) \times F_{ES-DD}(t) + f_{CR}(t) \times F_{CR-DD}(t)$$
$$+ f_{DD}(t) \times F_{DD-DD}(t) \quad \text{- Eq (14)}$$

The system has the following constraints:

a) $0 \leq F_{i-i}(t) \leq 1$, for *i = CB, ES, CR, DD;*

b) $0 \leq F_{i-DD}(t) \leq 1$, for *i = CB, ES, CR;*

c) $F_{i-i} + F_{i-DD} = 1$, for *i = CB, ES, CR*;

d) $F_{DD-DD}(t) = 1$

e) $0 \leq q_i(t) \leq 1$, for *i = CB, ES, CR*.

All other notations used in section S2 remain the same.

Eq (11-14) can be written in matrix format as follows,

$$\begin{bmatrix} fd \times f_{CB} \\ fd \times f_{ES} \\ fd \times f_{CR} \\ fd \times f_{DD} \end{bmatrix}_{t+\Delta t} = \begin{bmatrix} F_{CB-CB} + q_{CB} & 0 & 0 & 0 \\ 0 & F_{ES-ES} + q_{ES} & 0 & 0 \\ 0 & 0 & F_{CR-CR} + q_{CR} & 0 \\ F_{CB-DD} & F_{ES-DD} & F_{CR-DD} & 1 \end{bmatrix}_t \times \begin{bmatrix} f_{CB} \\ f_{ES} \\ f_{CR} \\ f_{DD} \end{bmatrix}_t$$

Following the constraint $F_{i-i} + F_{i-DD} = 1$, for *i = CB, ES, CR*, we do not need to separately estimate the fractional flow of cells to dead state. Therefore, the above matrix can be reduced to,

$$\begin{bmatrix} fd \times f_{CB} \\ fd \times f_{ES} \\ fd \times f_{CR} \end{bmatrix}_{t+\Delta t} = \left[\begin{bmatrix} F_{CB-CB} & 0 & 0 \\ 0 & F_{ES-ES} & 0 \\ 0 & 0 & F_{CR-CR} \end{bmatrix}_t + \begin{bmatrix} q_{CB} & 0 & 0 \\ 0 & q_{ES} & 0 \\ 0 & 0 & q_{CR} \end{bmatrix} \right] \times \begin{bmatrix} f_{CB} \\ f_{ES} \\ f_{CR} \end{bmatrix}_t$$

This can be written in vector notion as

$$\boldsymbol{C}(t + \Delta t) = [\boldsymbol{A}(t) + \boldsymbol{Q}(\boldsymbol{t})] \times \boldsymbol{B}(t) \qquad \text{- Eq (15)}$$

For this null model, we estimate the ***A(t)*** and ***Q(t)*** following the method described in S2.2

Supplementary Table S1: **Estimated fractional cell division values**

Time interval (h)		Untreated	1 ng/mL EGF	5 ng/mL EGF	10 ng/mL EGF	25 ng/mL EGF
0-12	q_{CB}	0.174	0.198	0.093	0.023	0
	q_{ES}	0.174	0.198	0.093	0.023	0
	q_{CR}	0.174	0.198	0.093	0.023	0
12-24	q_{CB}	0.139	0.127	0.164	0.095	0.024
	q_{ES}	0.139	0.127	0.165	0.095	0.024
	q_{CR}	0.139	0.127	0.164	0.095	0.024
24-36	q_{CB}	0.082	0.059	0.024	0.087	0.051
	q_{ES}	0.082	0.059	0.024	0.087	0.051
	q_{CR}	0.082	0.059	0.024	0.087	0.051
36-48	q_{CB}	0	0	0.012	0.012	0
	q_{ES}	0	0	0.012	0.012	0
	q_{CR}	0	0	0.012	0.012	0
48-60	q_{CB}	0.012	0.037	0.025	0	0
	q_{ES}	0.012	0.037	0.025	0	0
	q_{CR}	0.012	0.037	0.025	0	0

Supplementary Table S2: Estimated fractional state transition values ($F_{i\text{-}j}$):

a) Untreated:

	0-12 h				12-24 h				24-36 h			
	CB	ES	CR	DD	CB	ES	CR	DD	CB	ES	CR	DD
CB	0.78	0.12	0.09	0.01	0.93	0.04	0.03	0	0.93	0.04	0.03	0
ES	0.24	0.33	0.17	0.26	0.34	0.29	0.17	0.2	0.34	0.28	0.23	0.15
CR	0.35	0.28	0.35	0.02	0.35	0.41	0.21	0.03	0.21	0.59	0.19	0.01

	36-48 h				48-60 h			
	CB	ES	CR	DD	CB	ES	CR	DD
CB	0.85	0.08	0.06	0.01	0.92	0.03	0.05	0
ES	0.33	0.29	0.23	0.15	0.33	0.33	0.22	0.12
CR	0.2	0.67	0.06	0.07	0.21	0.67	0.05	0.07

b) 1 ng/mL EGF:

	0-12 h				12-24 h				24-36 h			
	CB	ES	CR	DD	CB	ES	CR	DD	CB	ES	CR	DD
CB	0.68	0.22	0.08	0.02	0.67	0.22	0.08	0.03	0.75	0.13	0.11	0.01
ES	0.7	0.16	0.11	0.03	0.73	0.14	0.09	0.04	0.72	0.14	0.12	0.02
CR	0.57	0.07	0.32	0.04	0.57	0.09	0.29	0.05	0.44	0.17	0.29	0.1

	36-48 h				48-60 h			
	CB	ES	CR	DD	CB	ES	CR	DD
CB	0.89	0.06	0.04	0.01	0.72	0.1	0.16	0.02
ES	0.59	0.25	0.13	0.03	0.47	0.29	0.23	0.01
CR	0.45	0.17	0.2	0.18	0.4	0.18	0.22	0.2

Supplementary Table S2 continued.

c) 5 ng/mL EGF:

	0-12 h				12-24 h				24-36 h			
	CB	ES	CR	DD	CB	ES	CR	DD	CB	ES	CR	DD
CB	0.14	0.06	0.8	0	0.3	0.15	0.32	0.23	0.81	0.14	0.04	0.01
ES	0.31	0.42	0.13	0.14	0.32	0.43	0.12	0.13	0.34	0.43	0.12	0.11
CR	0.63	0.26	0.05	0.06	0.66	0.32	0.02	0	0.77	0.2	0.02	0.01

	36-48 h				48-60 h			
	CB	ES	CR	DD	CB	ES	CR	DD
CB	0.84	0.12	0.04	0	0.87	0.08	0.04	0.01
ES	0.34	0.44	0.14	0.08	0.35	0.45	0.17	0.03
CR	0.77	0.19	0.03	0.01	0.73	0.2	0.03	0.04

d) 10 ng/mL EGF:

	0-12 h				12-24 h				24-36 h			
	CB	ES	CR	DD	CB	ES	CR	DD	CB	ES	CR	DD
CB	0.07	0.07	0.86	0	0.07	0.11	0.77	0.05	0.56	0.18	0.11	0.15
ES	0.5	0.25	0.11	0.14	0.5	0.26	0.11	0.13	0.65	0.26	0.04	0.05
CR	0.32	0.09	0.5	0.09	0.31	0.1	0.56	0.03	0.61	0.3	0.07	0.02

	36-48 h				48-60 h			
	CB	ES	CR	DD	CB	ES	CR	DD
CB	0.7	0.18	0.11	0.01	0.78	0.12	0.09	0.01
ES	0.65	0.26	0.04	0.05	0.62	0.27	0.04	0.07
CR	0.55	0.28	0.08	0.09	0.51	0.27	0.08	0.14

Supplementary Table S2 continued.

e) 25 ng/mL EGF:

	0-12 h				12-24 h				24-36 h			
	CB	ES	CR	DD	CB	ES	CR	DD	CB	ES	CR	DD
CB	0.07	0.09	0.81	0.03	0.04	0.04	0.91	0.01	0.04	0.03	0.91	0.01
ES	0.07	0.14	0.56	0.23	0.07	0.14	0.56	0.23	0.08	0.29	0.38	0.25
CR	0.1	0.17	0.7	0.03	0.08	0.15	0.75	0.02	0.11	0.14	0.74	0.01

	36-48 h				48-60 h			
	CB	ES	CR	DD	CB	ES	CR	DD
CB	0.07	0.05	0.75	0.13	0.09	0.05	0.74	0.12
ES	0.06	0.46	0.3	0.19	0.05	0.49	0.24	0.22
CR	0.09	0.09	0.79	0.02	0.15	0.15	0.69	0

Supplementary Table S3: Antibodies used in the experiments

a) Western blotting:

Antibody	Make	Dilution
Anti-Phospho-EGF Receptor (Tyr 1068)	Cell Signaling Technology-3777	1:4000
Anti-EGF Receptor	Cell Signaling Technology-4267	1:4000
Anti-FAK	Cell Signaling Technology-13009	1:2000
Anti-Phospho-FAK (Tyr 397)	Invitrogen-700255	1:2000
Goat anti-rabbit HRP-conjugate	Cell Signaling Technology-7074P2	1:5000

Supplementary Table S3 continued.

b) Immunofluorescence:

Antibody	Make	Dilution
Anti-Vimentin Alexa Fluor 488 conjugated	Abcam-ab195877	1:50
Anti-Snail1 Alexa Fluor 488 conjugated	eBioscience-53-9859-82	1:50

c) Flow cytometry:

Antibody	Make	Dilution
Anti-Phospho-EGF Receptor (Tyr 1068)	Cell Signaling Technology-3777	1:500
Anti-EGF Receptor	Cell Signaling Technology-4267	1:500
Goat anti-Rabbit Alexa Fluor 488 conjugated	Invitrogen-A-11070	1:1000

Supplementary Table S4: Primers used in qPCR

Gene		Sequence
Vimentin	Forward	AGTCCACTGAGTACCGGAGAC
	Reverse	CATTTCACGCATCTGGCGTTC
Fibronectin	Forward	AGGAAGCCGAGGTTTTAACTG
	Reverse	AGGACGCTCATAAGTGTCACC
Snail 1	Forward	TCGGAAGCCTAACTACAGCGA
	Reverse	AGATGAGCATTGGCAGCGAG
Zeb 1	Forward	TTACACCTTTGCATACAGAACCC
	Reverse	TTTACGATTACACCCAGACTGC

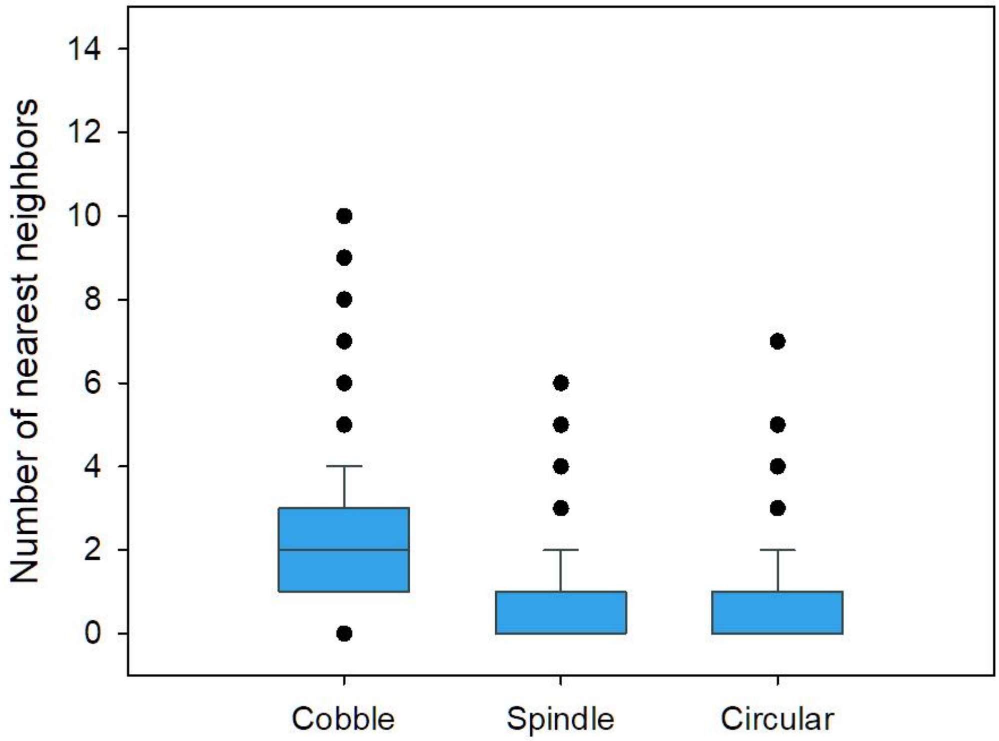

Supplementary Figure S2: Spindle and Circular cells are more scattered than cobble cells. MDA-MB-468 cells were treated with 10 ng/mL of EGF and number of nearest neighbours for each cell was measured by image analysis. A radial distance of five pixels from the periphery of each cell is defined as the neighbouring distance to that particular cell. Cells lying within the neighbouring distance of a cell are the nearest neighbours to that particular cell. The differences in the median numbers of nearest neighbours of three cell types are statistically significant (Kruskal-Wallis test, $p < 0.01$).

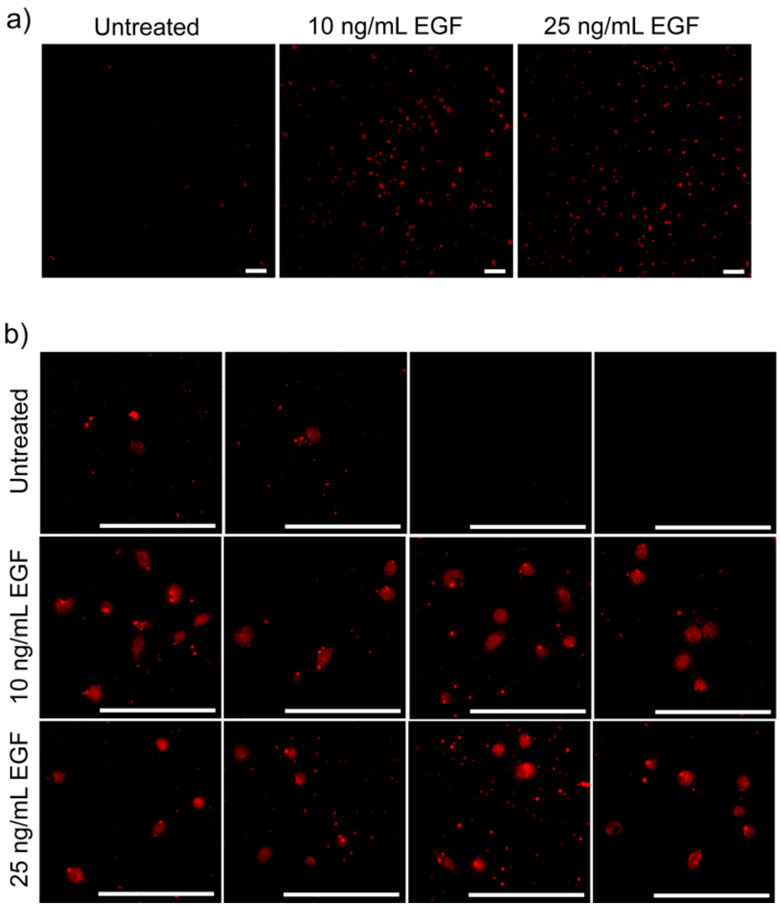

Supplementary Figure S3: Migration assay. Cells were treated with different doses of EGF for 24 hr and migration was assayed using Boyden Chamber. Cells migrated to the other side of the transwell membrane were stained and imaged. a) The membrane was scanned with the 5X objective lens. Each image represents one of the individual tiles scanned from the entire membrane. b) Four randomly zoomed-in locations from the images in S3a to show the shape of cells with clarity. Scale bar: 200 μm.

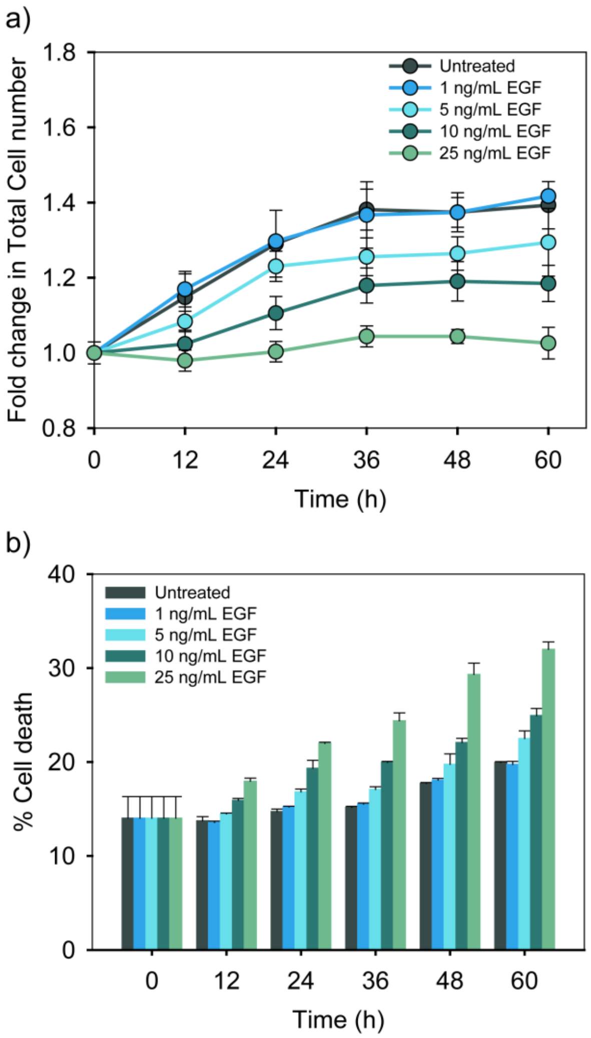

Supplementary Figure S4: Effect of EGF on cell death and viability. MDA-MB-468 cells were treated with different doses of EGF for different durations. (a) Fold change in total cell number and (b) the percentage of cell death, were measured by Propidium Iodide assay. Each data point represents the mean of three independent assays and error bars indicate standard deviation. Fold change in cell number with time was statistically significant only for untreated, 1 ng/mL, 5 ng/mL and 10 ng/mL EGF treated cells (Kruskal-Wallis Analysis of Variance $p < 0.05$). There was no statistically significant difference between untreated and 1 ng/mL EGF treated cells. Time-dependent increase in cell death was statistically significant in all treatment groups (Analysis of variance, $p < 0.01$).

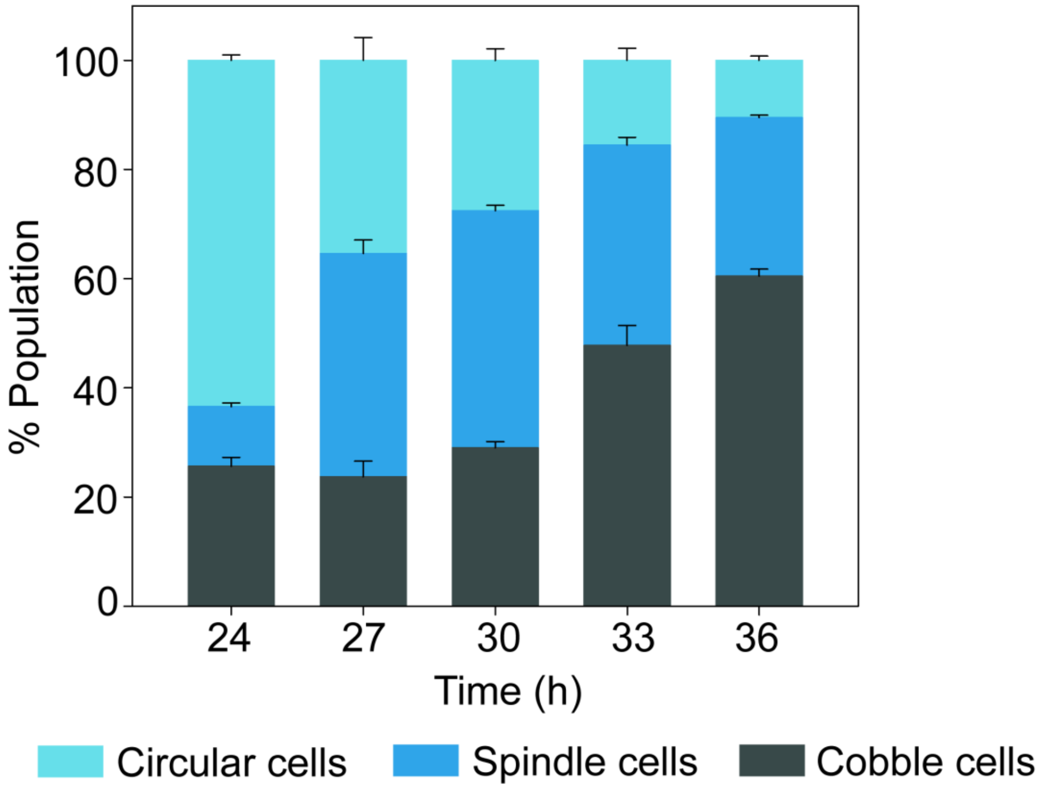

Supplementary Figure S5: Population distribution of MDA-MB-468 cells when treated with 10 ng/mL of EGF and imaged at 3 h intervals in the period of 24 to 36 h. Each data point represents the mean of three independent experiments and error bars indicate standard deviation.

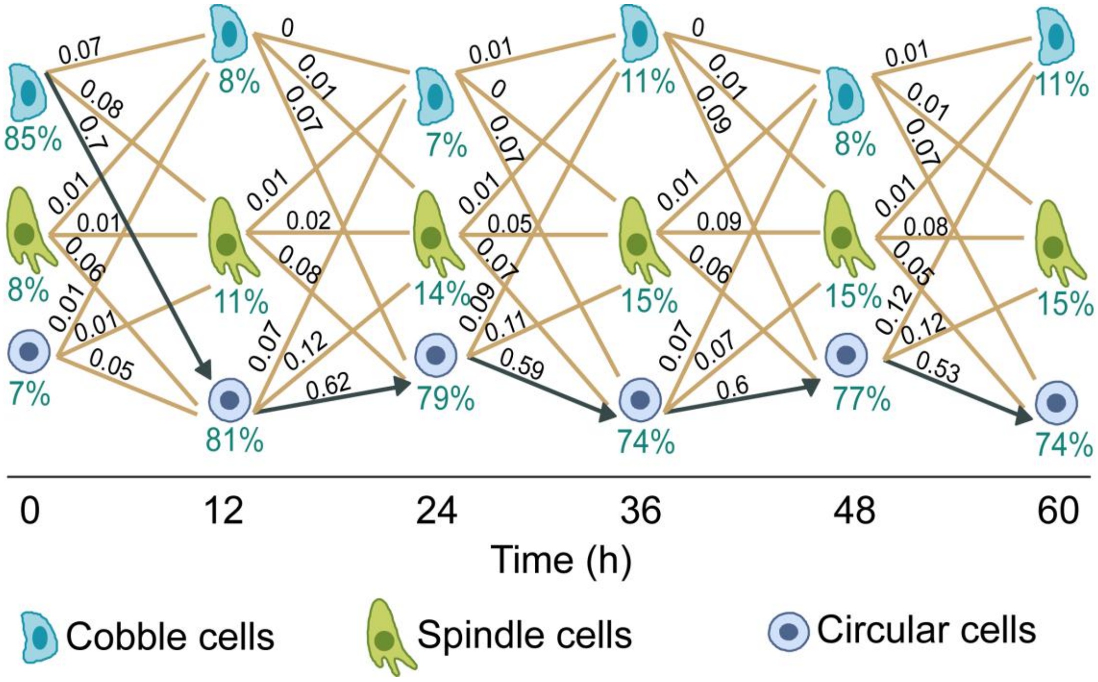

Supplementary Figure S6: Cell state transition diagram for MDA-MB-468 cells treated with 25 ng/mL of EGF. Each line represents one state transition path. State transition parameters were estimated from the quantitative imaging data. Numerical values over the lines indicate the normalized flow of cells through these paths. Pointed black arrows show the dominant transition paths.

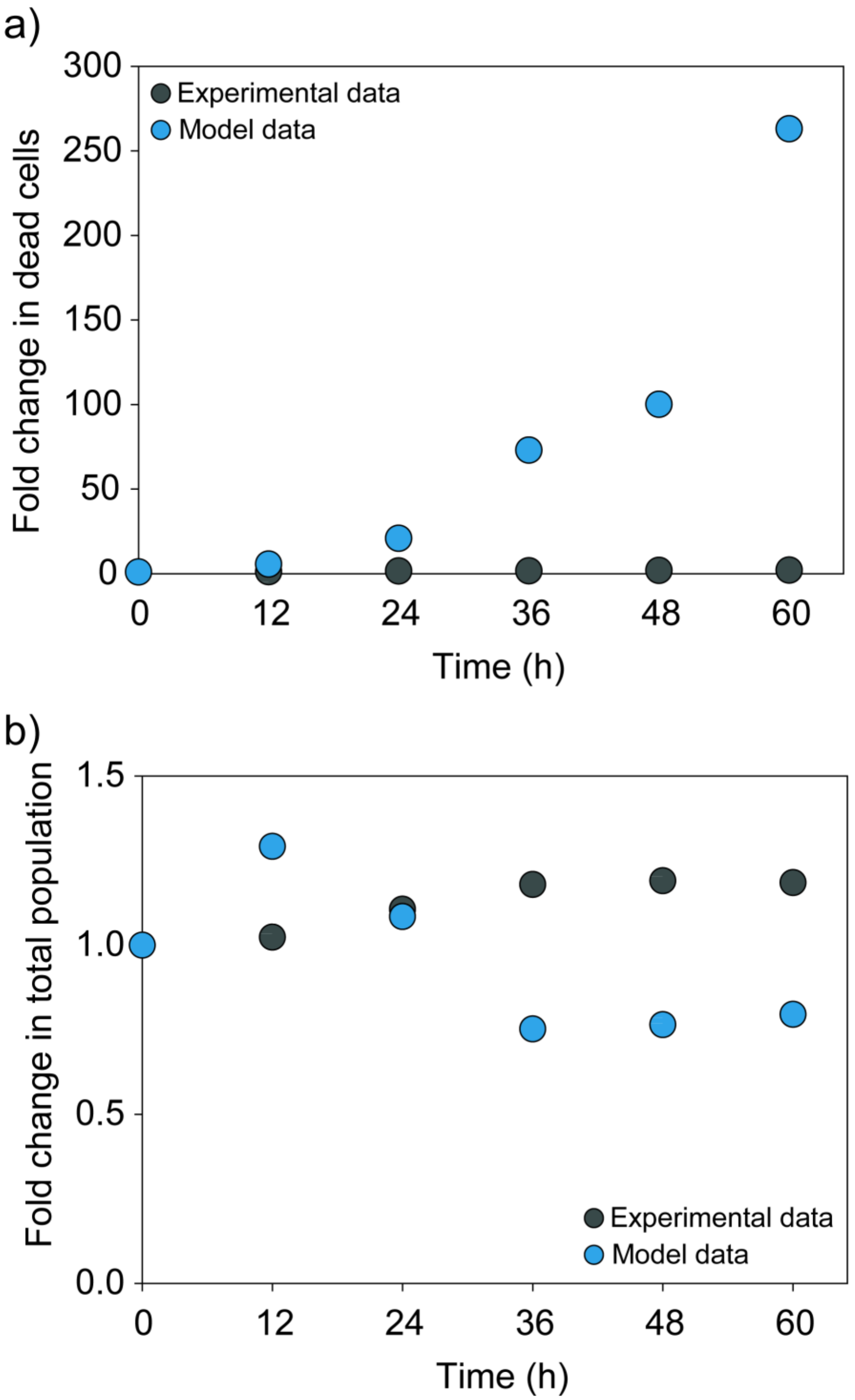

Supplementary Figure S7: Comparison between experimental observations and predictions by the null model. a) Fold change in the number of dead cells and b) fold change in the total cell number for cells treated with 10 ng/mL EGF.

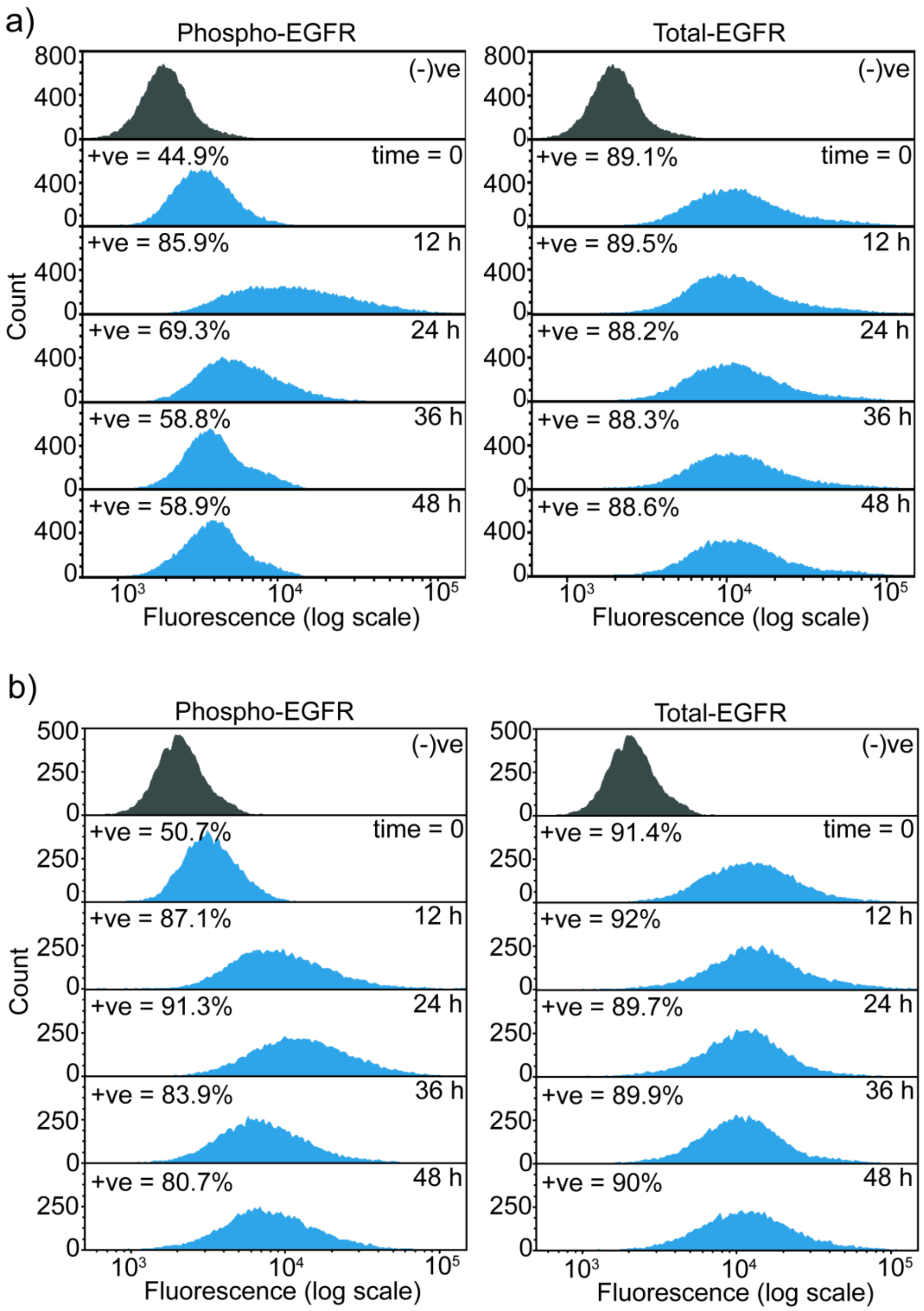

Supplementary Figure S8: Dynamics of phospho-EGFR and total EGFR measured by flow cytometry. a) 10 ng/mL of EGF treated cells. b) 25 ng/mL of EGF treated cells. Percentage positive population was estimated through Overton histogram subtraction. Cells stained with only secondary antibody was used as a control in histogram subtraction (-ve control).

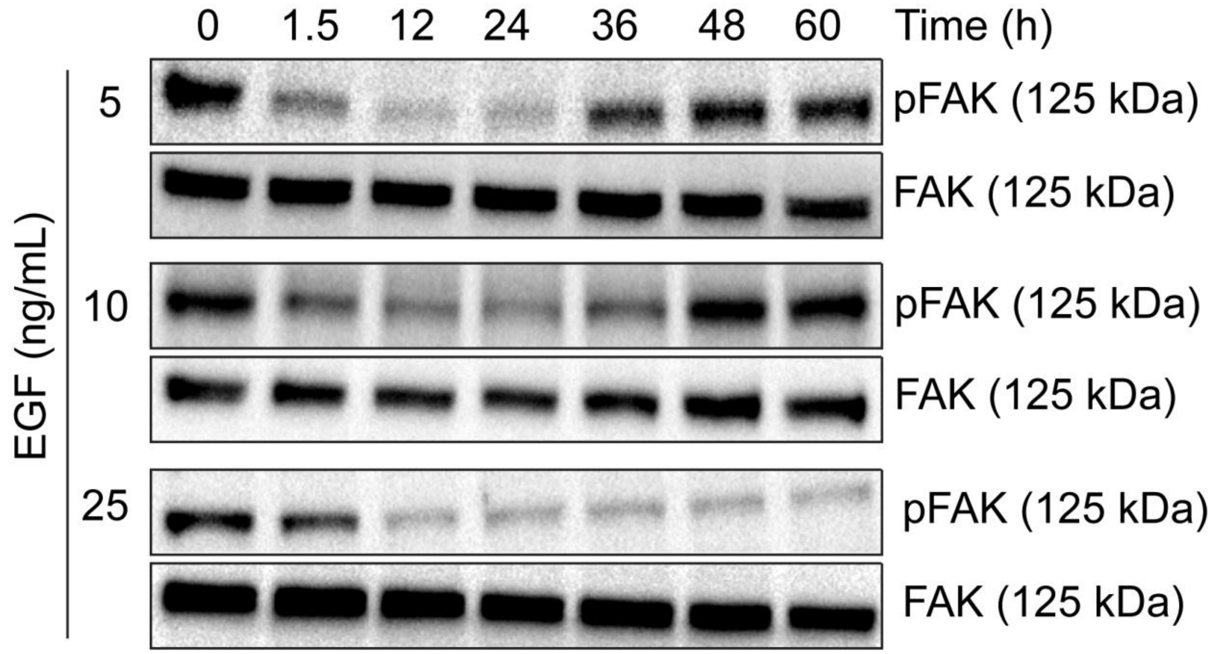

Supplementary Figure S9: Temporal dynamics of phosphorylation of FAK. MDA-MB-468 cells were treated with different doses of EGF and different molecules were detected by Western Blots.

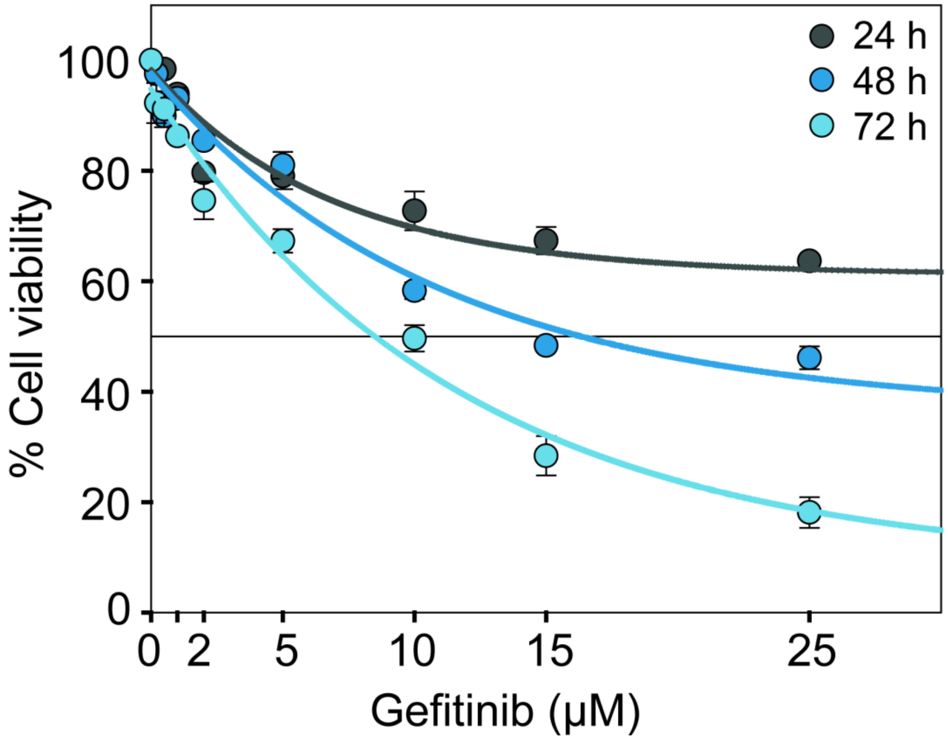

Supplementary Figure S10: Effect of Gefitinib on the viability of cells. MDA-MB-468 cells were treated with different doses of Gefitinib for different durations and cell viability was measured by MTT assay. The percentage cell viability was calculated relative to cells treated with an equivalent amount DMSO in media (without Gefitinib). Dose- and time-dependent effect of Gefitinib on cell viability was statistically significant (two-way ANOVA, $p < 0.01$).

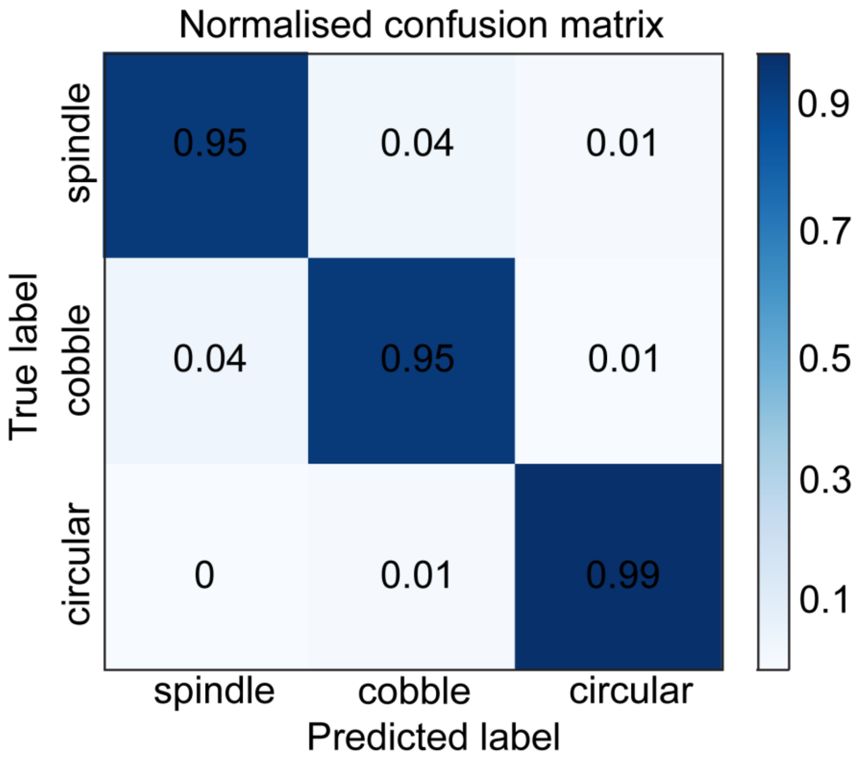

Supplementary Figure S11: The plot shows the quality of cell type classification through machine learning. True label denotes the actual cell type defined by the user. Predicted label denotes the cell type identified by the algorithm after machine learning. Diagonal elements represent the correct prediction of the cell type and the non-diagonal elements represent the false positives. A value of 1 indicates that all the cells were rightly classified by the machine as defined by the user.

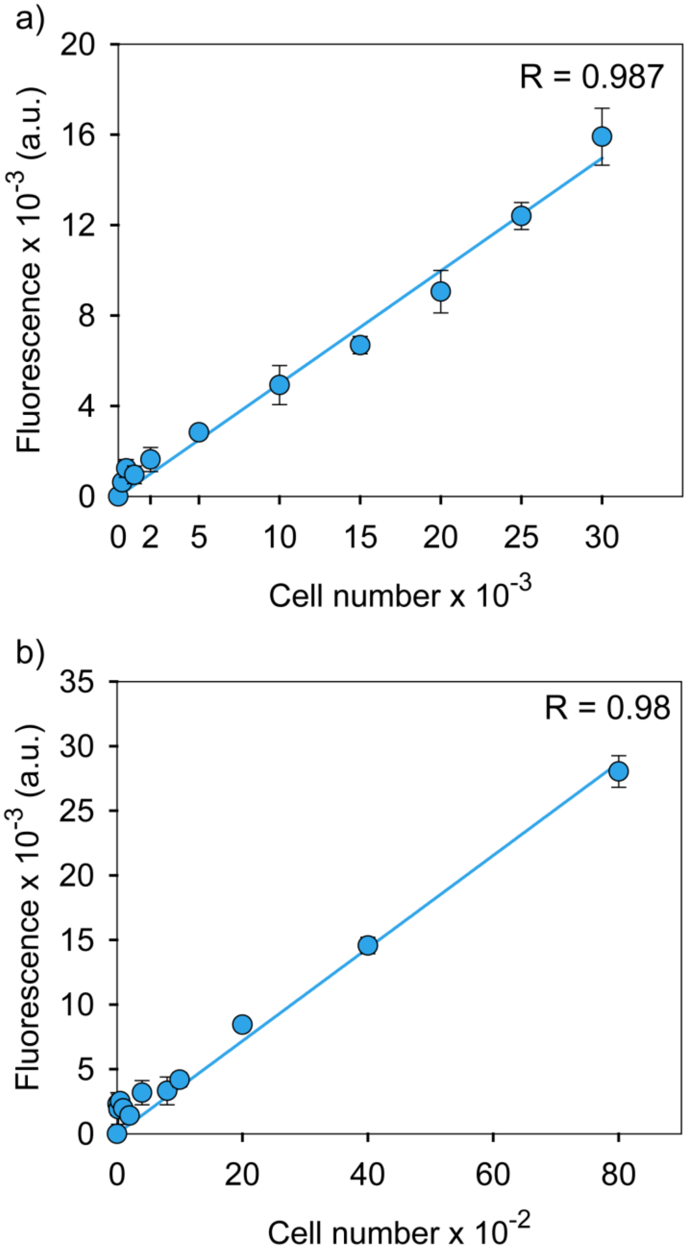

Supplementary Figure S12: Standard curve to determine the linear regime of fluorescence-based plate reader assay. a) The linear regime of total cell number measurement assay. b) The linear regime of dead cell number measurement assay. R: correlation coefficient for linear regression.

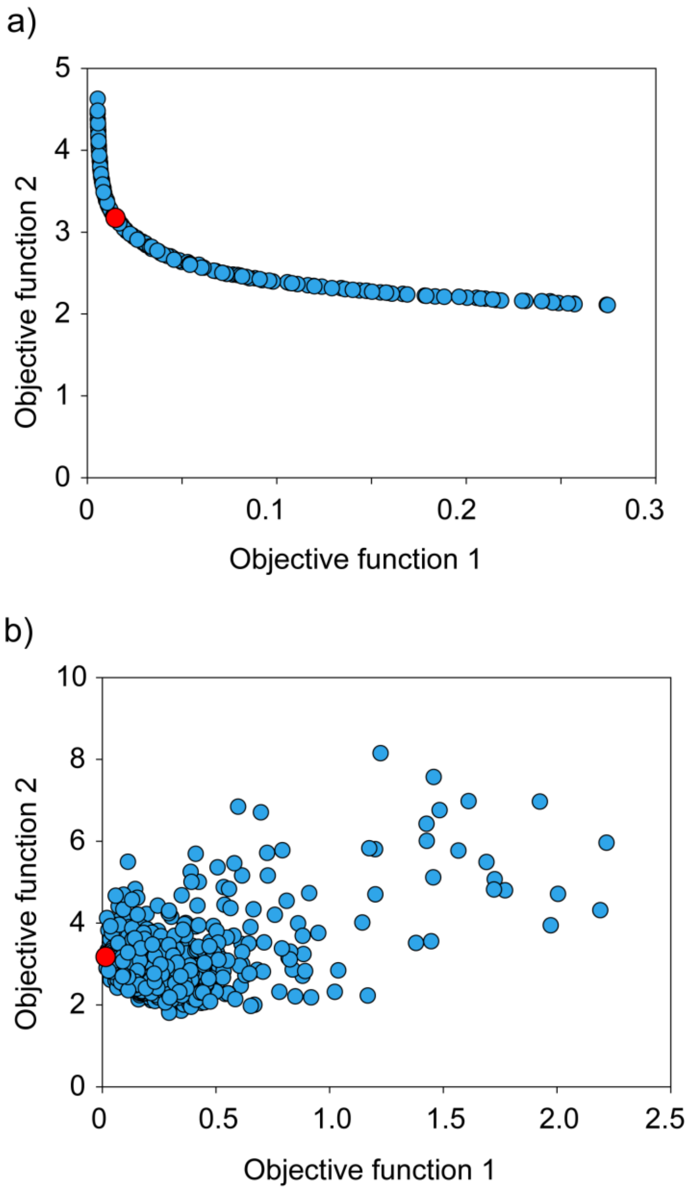

Supplementary Figure S13: a) Pareto front of one of the 1000 independent parameter estimations for 10 ng/mL EGF treated cells. Red filled circle represent the optimal solution of the Pareto front. b) Distribution of the objective functions. Each blue filled circle represents the best solution of each of the 1000 independent parameter estimations for 10 ng/mL EGF treated cells. Red filled circle represent the overall optimal solution.

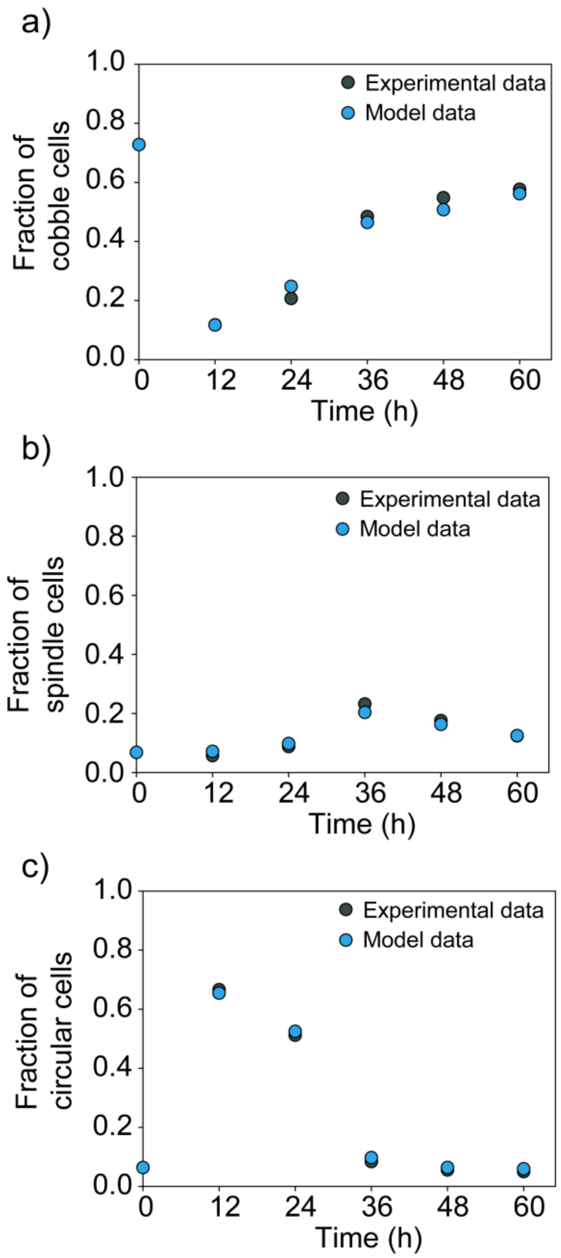

Supplementary Figure S14: Comparison between experimental observations and predictions by the state-transition model for cells treated with 10 ng/mL of EGF. a) for cells is Cobble state, b) for cells in spindle state and c) for cells in circular state.

References

1. Coleman, T.F.; Li, Y. A reflective Newton method for minimizing a quadratic function subject to bounds on some of the variables. *SIAM Journal on Optimization* **1996**, *6*, 1040-1058.
2. Chiba, T.; Hino, H.; Akaho, S.; Murata, N. Time-varying transition probability matrix estimation and its application to brand share analysis. *PLoS ONE* **2017**, *12*.
3. Deb, K.; Kalyanmoy, D. *Multi-Objective Optimization Using Evolutionary Algorithms*; John Wiley \& Sons, Inc.: 2001; pp. 518.
4. Deb, K.; Gupta, S. Understanding knee points in bicriteria problems and their implications as preferred solution principles. *Engineering Optimization* **2011**, *43*, 1175-1204.
